# Comparison of Growth Performance and Biochemical Components between Low-Salinity-Tolerant Hybrid and Normal Variety of Pacific White Shrimp (*Penaeus vannamei*)

**DOI:** 10.3390/ani13182837

**Published:** 2023-09-07

**Authors:** Yucong Ye, Bihong Zhu, Junya Zhang, Ying Yang, Jiangtao Tian, Wenyue Xu, Xinglin Du, Yizhou Huang, Yiming Li, Yunlong Zhao

**Affiliations:** 1 School of Life Science, East China Normal University, Shanghai 200241, China; ycy_666@yeah.net (Y.Y.); 51251200028@ecnu.edu.cn (B.Z.); 18326810369@163.com (J.Z.); yangying_ecnu@163.com (Y.Y.); tjttony@126.com (J.T.); 51211300037@stu.ecnu.edu.cn (W.X.); duxinglin-1@163.com (X.D.); hyz20180925@163.com (Y.H.); 2 Fishery Machinery and Instrument Research Institute, Chinese Academy of Fisheries Sciences, Shanghai 200092, China; 3 State Key Laboratory of Estuarine and Coastal Research, East China Normal University, Shanghai 200241, China

**Keywords:** *Penaeus vannamei*, growth performance, biochemical composition, gene expression

## Abstract

**Simple Summary:**

The Pacific white shrimp (*Penaeus vannamei*), a high-yielding economic shrimp, is facing germplasm degradation in the freshwater aquaculture environment in China, resulting in a drastic decline in production. We used hybridization to obtain shrimp with low-salinity-tolerant characteristics. A 90-day growth comparison experiment was conducted using a low-salinity-tolerant hybrid population and a normal variety population, and it was found that the low-salinity-tolerant hybrid population was superior to the normal variety population in terms of survival, growth performance and nutrient content. Our study provides a valuable reference for subsequent genetic breeding and shrimp culture.

**Abstract:**

*Penaeus vannamei*, a high-yield economical shrimp, is confronting germplasm degradation in the culture environments found in China, which results in a sharp drop in production. Genetic improvement by hybridization is an effective way to solve this problem. In this study, we selected the hybrid species adapted to low-salinity culture obtained by intraspecific crossing as the experimental group. The control group consisted of normal variety from the Hainan Lutai Company. The two groups of shrimps were cultured for three months under salinities of 1 PSU, 5 PSU, and 15 PSU. Growth-performance-related indicators, biochemical composition, and molting-related gene expression were examined. The results showed that at salinities of 1 PSU and 5 PSU, the survival rate and growth performance of the low-salt breeding group were better than those of the normal variety population. The digestive enzyme activity in the low-salt breeding group was higher, which was consistent with its better growth performance, and was also associated with higher triglyceride, total cholesterol, and glycogen content. Lower levels of lactic acid indicated less anaerobic metabolism and better adaptability to the environment. The amino acid and fatty acids analysis showed that levels of essential amino acids and high unsaturated fatty acids were both higher in the low-salt breeding group than in the normal variety shrimp cultured in a low-salinity environment. The expression levels of genes associated with molting (*CHS*, *CaMKI*, *RXR*, *EcR*, *HSP60,* and *HSP70*) were also higher in the low-salt breeding group than in the control group. The results indicated that the hybrid shrimp showed better growth performance and nutritional advantages compared with the normal shrimp under salinities of 1 PSU and 5 PSU. This research provides a valuable reference for subsequent genetic breeding and shrimp culture.

## 1. Introduction

*Penaeus vannamei*, also known as Pacific white shrimp or white-leg shrimp, is the most economically valuable cultured shrimp in the world [1]. It is distributed along the Pacific coast of Central and South America, has been introduced to the Eastern hemisphere, and has become the primary species currently being cultured in Southeast Asian countries [2]. A growing world population and increasing demand for protein have resulted in the development of fisheries globally [3]. *P. vannamei* is a major aquaculture species that exhibits a rapid growth rate and tolerance to a wide range of environmental conditions such as temperature and salinity [4]. Because of its high yield, *P. vannamei* was introduced into China for breeding and now is the largest variety of shrimp raised in China [5]. In 2021, the farming volume of *P. vannamei* accounted for 46% of the total shrimp farming in China [6]. Cultures of marine shrimp species are primarily conducted in coastal areas with estuarine and oceanic water. However, in the past decade, the expansion of euryhaline shrimp production has moved away from the coastal environment to inland waters to take advantage of the freshwater resources and promote economic development in some inland regions [7].

Although *P. vannamei* is a euryhaline shrimp species and can tolerate a wide range of salinity from 1 to 50 practical salinity units (PSU) [8], the growth of shrimp at a salinity of 1–2 PSU was significantly less than that at a salinity of 4–5 PSU [9]. The specific growth rate, food consumption, food use efficiency, and absorption efficiency were highest in shrimp at salinities of 15–20 PSU [10]. With the development of freshwater aquaculture, also with the desire of meeting the increasing demand for shrimp as food, aquaculturists have shifted from an extensive system of culturing to semi-intensive and intensive systems [11]. However, there are a series of problems such as the deterioration of the aquaculture water environment, the frequent occurrence of diseases, and the abuse of aquatic drugs [12]. In addition, large-scale high-density culture has led to the intensification of inbreeding, resulting in the degradation of germplasm resources [13]. Research shows that inbreeding affects the reproductive characteristics of shrimp [14]. Therefore, the *P. vannamei* aquaculture industry is facing severe challenges [15]. Among these problems, the frequent occurrence of disease and the abuse of aquatic drugs have been widely studied, but the degradation of germplasm resources has not been resolved.

It is a good method to improve the productivity of the cultured population by exploiting the potential advantages in the hybrid offspring (hybrid vitality) through crossbreeding [16]. Hybridization (conclude interspecific, intraspecific, and involve subspecies) increases the genetic variation of a population, including genotypic and phenotypic variation in the offspring, which plays an important role in adaptive evolution [17,18]. Studies have shown that a novel hybrid (*Hypophthalmichthys nobilis* (2n = 48 ♀) × *Megalobrama amblycephala* (2n = 48 ♂)) had the characteristics of a herbivore with fast growth [19]. Zhao et al. [20] reported that sea urchin hybrids (*Heliocidaris crassispina* ♀ *× Strongylocentrotus intermedius* ♂) could be a prospective aquaculture urchin species with higher nutrient content, greater medicinal value, and improved flavor. Intraspecific hybridization of two geographically distinct populations of Kumamoto oysters (*Crassostrea sikamea*) produces progeny with high survival and rapid growth rates [21]. Therefore, an urgent task is to obtain *P. vannamei* with growth advantages in a freshwater environment by intraspecific hybridization.

The growth of aquatic animals is closely related to the digestive function, and digestion is the main physiological process for animals to obtain energy and nutrients [22]. The hepatopancreas is an important organ for crustaceans involved in digestion, absorption, and growth, and it is also a protective barrier against external stress [23]. The main functions of the hepatopancreas include food absorption, transport, digestive enzyme secretion, and lipid storage [24]. Digestion is the main physiological process for animals to obtain energy and nutrition, involving the activation, recognition, and hydrolysis of food molecules at a specific time [22]. The activities of digestive enzymes (such as amylase, lipase, trypsin, and protease) have an impact on the growth performance of *P. vannamei* [25]. Likewise, triglycerides and total cholesterol are related to animal health [22]. Lactic acid is a by-product and an important participant in glucose metabolism. Moreover, the increased metabolic capacity of the organism leads to an increase in glucose and lactic acid levels in the body (Long et al., 2021 [26]). In addition, since the maintenance of ion homeostasis, normal cell function, and other physiological processes in aquatic animals require a lot of energy, at low salinity they may require more energy for osmoregulation to maintain body homeostasis, leading to less energy for shrimp growth [27].

Amino acid digestion and utilization are closely related to shrimp culture [28]. Essential amino acids (EAAs), as they cannot be synthesized by humans, must be obtained from the regular diet [29]. Studies have shown that leucine increased the content of muscle crude protein, increased the number and area of hepatopancreas fiber cells, and improved the growth performance of *P. vannamei* [30]. Besides, free amino acids can accumulate in intracellular compartments and have been shown to play an important role as organic penetrants in osmoregulation of marine invertebrates [31]. It was found that alanine and serine can help to regulate osmosis during salinity regulation of *P. vannamei* larvae [32]. Therefore, the composition of amino acids is very important for understanding the growth and regulation of shrimp under low salt.

Lipid metabolism plays an important role in the response of aquatic animals to changes in environmental salinity [33]. Fatty acids, as an important part of lipid metabolism, are essential nutrients in aquatic organisms and play an important role in embryo development, early larva development, and growth [34]. Individual fatty acids consist of saturated fatty acids (SFAs), monounsaturated fatty acids (MUFAs), polyunsaturated fatty acids (PUFAs), and highly unsaturated fatty acids (HUFAs). In aquatic animals, tissue LC-PUFA contents fluctuate in response to environmental salinities [35]. Therefore, the composition and proportion of fatty acids are very important for reflecting the osmotic ability of aquatic organisms.

Molting is an important process necessary for the continued growth of crustaceans, which is considered to be the most sensitive of the stages to environmental stressors [36,37]. Cholesterol is an essential component of all animal cell membranes and functions, and its levels are affected by environmental factors, including temperature, salinity, and pollutants [38]. Ecdysteroids, by binding to the ecdysteroid receptor (EcR) and retinoid X receptor (RXR), promote the degradation of the old exoskeleton and the formation of the new one [22]. The role of chitin synthase (CHS) in the ecdysis process is to recover the hydrolysate of the old exoskeleton and synthesize the new exoskeleton [39]. Molting is a necessary way for crustaceans to grow, and low salinity has a significant effect on the growth of *P. vannamei* [33]. Hybridization results in *Macrobrachium nipponense* show that heterosis can significantly increase the expression of molting-related genes, thus promoting growth [22]. Therefore, understanding the molting of prawns is also helpful to elucidate the molecular mechanism of heterosis during the growth of low-salt-tolerant hybrid populations.

In this study, a hybrid with low salt tolerance was obtained by using a combination of double-cross and family breeding strategies. By comparing the growth performance of *P. vannamei* bred with a low-salt family with that of a normal variety population under different salinities, the growth characteristics of the *P. vannamei* hybrid were determined. Therefore, this study compared the growth performance of the low-salt breeding group and the normal variety shrimp group under low salt stress, to provide alternative strategies in *P. vannamei* aquaculture.

## 2. Material and Methods

### 2.1. Populations and Experimental Families

**Figure 1:**
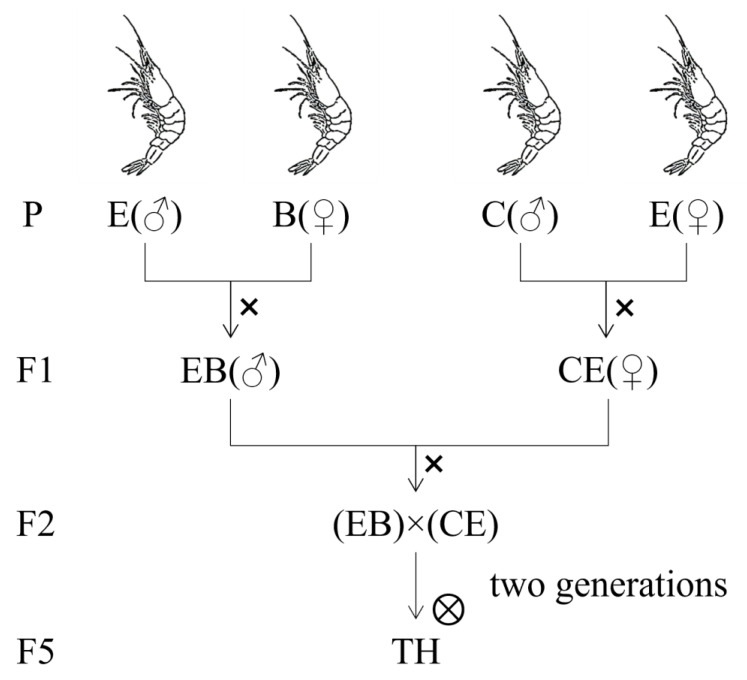
The two-line hybridization strategy and family selection strategies yielded low salt tolerance of *Penaeus vannamei*.

The shrimp in this experiment were from Shanghai Ocean University. The parent populations were three wild populations (B, C, E) from Guam, which had the advantages of freshwater adaptation or fast growth. These three populations were made intraspecific crosses, and the F1 (EB and CE) was obtained through pedigree selection. Two generations of self-cross, EB♂ × CE♀, were obtained, which could grow fast and were suitable for freshwater breeding. The low-salt-tolerance shrimp bred by the double-cross and family strategies were selected as the hybrid breeding group (TH), and shrimp of the normal variety from the Lutai Company (Wenchang, China) were selected as the control group (TC). The hybridization strategy is shown in Figure 1.

### 2.2. Experimental Animal Culture

The juvenile shrimp were acclimated for 2 weeks before the experiment began. The formal experiment was a 12-week culture experiment. A literature review showed that the suitable growth salinity of *P. vannamei* was 15 PSU, the salinity of 5 PSU was the desalination culture salinity commonly used, and the salinity of 1 PSU was low-salt-stress salinity [10,40]. Therefore, three different salinities (1 PSU, 5 PSU, and 15 PSU) were selected to set the low-salt breeding group (TH) and the normal variety shrimp group (TC). Each gradient and each group were set with three replicates, totaling 18 aquariums of 60 L. Fifty shrimp were placed in each aquarium and fed commercial feed (Haida Feed, Guangzhou, Guangdong, China) containing 5% of the total weight of the shrimp three times (7:00, 15:00, 23:00) every day. Half of the water was changed every two days, and residual bait and dead shrimp were removed promptly. During the culture period, oxygenation was continued to ensure that dissolved oxygen was greater than 6.5 mg/L, the water temperature was maintained at 25 ± 2 °C, total ammonia nitrogen was less than 0.1 mg/L, and the pH was 8.1 ± 0.1.

The experimental procedures and animal care were according to the Committee on the Ethics of Animal Experiments in East China Normal University (f20201001) and the Care and Use of Laboratory Animals in China.

### 2.3. Sample Collection and Evaluation of Growth

During the experiment, nine shrimp were randomly selected from different salinities and genetic lines at 30, 60, and 90 days to determine body weight, hepatopancreas weight, and body length for calculation of the survival rate (SR), weight gain rate (WG), body-length growth rate (BGR), hepatosomatic index (HSI), condition factor (CF), gross feed conversion rate (GFCR), and specific growth rate (SGR). The shrimp were placed on ice to reduce their metabolism and manipulation stress, then weighed. Hepatopancreas and muscle tissue were completely removed from the shrimps using aseptic tweezers and scissors and the hepatopancreas was weighed to calculate the HSI. Tissues were placed in a centrifuge tube, frozen in liquid nitrogen, and stored at −80 °C. The formula for basic growth indicators is as follows:

SR (%) = (total shrimps − dead shrimps)/total shrimps × 100

WG (%) = (final weight − initial weight)/initial weight × 100

BGR (%) = (final body length − initial body length)/initial body length × 100

HSI (%) = (hepatopancreatic wet weight/body wet weight) × 100

CF (g/cm^3^) = body weight/(body length)^3^ × 100

GFCR (%) = feed dosage/(final weight − initial weight) × 100

SGR (%/day) = [(ln final weight − ln initial weight)/breeding days] × 100

Biochemical parameters and digestive enzyme activity analysis were performed using three hepatopancreas and muscles per group. Amino acid analysis was performed using three muscles per group. Fatty acid analysis was performed using three hepatopancreas per group. Gene expression analysis was performed using three hepatopancreas per group.

### 2.4. Biochemical Parameters Analysis

The biochemical parameters in the hepatopancreas and muscle were determined by commercial triglyceride, total cholesterol, and lactic acid kits (Nanjing Jiancheng Bioengineering Institute, Nanjing, China). All procedures were carried out according to the manufacturer’s instructions and results were obtained for biochemical components per gram of protein. In short, 0.1 g of tissue was collected, 0.9 mL of saline (homogenate medium, 0.86 g of NaCl dissolved in 100 g of water) was added, and after shaking and mixing, the samples were centrifuged at 2500 rpm and 4 °C for 20 min. Bradford reagent was used to quantify the protein content, and the amount of blue compound formed by the reaction was measured at 595 nm (enzyme activity was determined in the same way). The absorbance was measured using a Multiskan FC enzyme-labeling instrument (Thermo Fisher Scientific, Waltham, MA, USA). The concentrations of triglyceride, total cholesterol, and lactic acid were calculated by the standard curve of the substrate and separately measured at 540 nm, 500 nm, and 620 nm, respectively. In addition, glycogen content was determined according to the method previously described by Rosas et al. [41].

### 2.5. Digestive Enzyme Activity Analysis

The muscle and hepatopancreas tissues of each group were added to a sterilized centrifuge tube with 0.86% normal saline (homogenate medium, 0.86 g of NaCl dissolved in 100 g of water). After shaking and mixing, the samples were centrifuged at 2500 rpm and 4 °C for 20 min. Finally, the supernatant was collected for enzyme activity determination. The activities of the digestive enzyme in the hepatopancreas and muscle were determined by amylase, pepsin, trypsin, and lipase kits (Suzhou Comin Biotechnology Co., Ltd., Suzhou, China). All procedures were carried out according to the manufacturer’s instructions. The absorbance was measured using a Multiskan FC enzyme-labeling instrument (Thermo Fisher Scientific, Waltham, MA, USA). Amylase, pepsin, trypsin, and lipase separately measured at 540 nm, 580 nm, 555 nm, and 710 nm, respectively.

### 2.6. Amino Acid and Fatty Acid Analysis

The fatty acid composition of hepatopancreas tissues of shrimp was analyzed by gas chromatography–mass spectrometry (GC–MS). Each 100 mg sample (freeze-dried) was saponified first in KOH-methanol solution (1 mol/L) in a 65 °C water bath for 20 min and cooled on ice for 10 min. Fatty acid methyl esters (FAMEs) were synthesized in HCl-methanol (2 mol/L) solution in a 65 °C water bath for 20 min. After cooling on ice for 10 min, FAMEs were extracted in n-hexane, and the organic phase of the supernatant was analyzed by GC–MS (GSMS-QP 2010 SE, Shimadzu, Tokyo, Japan). The injector and detector temperatures were 250 °C and 300 °C, respectively. The column (DB-FFAP, Agilent, CA, USA) temperature was initially set at 150 °C for 1 min and finally increased to 220 °C at 6 °C min^−1^ and held for 15 min. The carrier gas was hydrogen (99.999% purity). FAMEs were identified by reference to a commercial standard (Sigma-Aldrich, St. Louis, MO, USA) and quantified using the area of each peak. 

For amino acid determination, about 0.2 g of shrimp muscle sample was first weighed, hydrolyzed with HCl (6 M), sealed with high-purity N_2_, and then placed in a constant-temperature air-dry oven at 110 °C for digestion. The hydrolysis product was cooled to room temperature, and 1 mL was evaporated to dryness in a water bath at 40 °C by adding ultrapure water. The sample was then resuspended using 1 mL of HCl (0.02 M). Samples were filtered using an ultrafiltration membrane (Millipore, Billerica, MA, USA) before analysis. Subsequently, 20 μL of sample solution was injected into the autosampler of a high-speed amino acid analyzer (LA8080, Hitachi High-tech Science, Marunouchi, Tokyo, Japan) for analysis.

### 2.7. Gene Expression Analysis

TRIzol reagent (Aidlab, Beijing, China) was used to extract total RNA from the hepatopancreas of the TH and TC shrimp cultured at different salt concentrations for 12 weeks (*n* = 3). The purity and content of the extracted RNA were measured using a Thermo NanoDrop 2000 instrument (Thermo Scientific, Wilmington, DE, USA, absorbance was measured at 260 nm and 280 nm (A260/280)) and 1% agarose gel electrophoresis. Total RNA was reverse-transcribed into first cDNA using a PrimeScript RT Master Mix Perfect Real-Time Kit (TaKaRa, Shiga, Japan). All procedures were completed in strict accordance with the instructions of the kit. The transcribed cDNA was stored at −20 °C.

Gene sequences were obtained from NCBI databases (https://www.ncbi.nlm.nih.gov/, accessed on 1 June 2023). Primer 5 software was used to design primers for the molting-related genes (*CaMKI, EcR, CHS, RXR, HSP60, HSP70*) and internal reference gene (*β-actin*) (Table 1). All primers were synthesized by Sangon Biotech Co., Ltd. (Shanghai, China). A SYBR qPCR master mixing kit (Vazyme Biotechnology, Nanjing, China) was used to evaluate the expression of growth-related genes. All fluorescence quantitative PCR experiments were performed on a real-time Bio-Rad CFX96 system (Hercules, CA, USA). The PCR reaction volume was 20 µL, containing 2 × ChamQ Universal SYBR qPCR main mixture (10 μL), 1μL of cDNA template, and 0.4 μL (10 μM) of forward and reverse primers. The reaction conditions were as follows: predenaturation at 95 °C for 30 s, 40 cycles of denaturation at 95 °C for 10 s, and annealing at 60 °C for 30 s. Three replicates were performed for each sample.

### 2.8. Statistical Analysis

Microsoft Office Excel 2021 and SPSS Statistics 23.0 were used for data analysis. All experimental data were expressed as mean ± standard deviation (SD). The significant differences among various groups were determined via a one-way ANOVA followed by Tukey’s test (*p* = 0.05). The 2^−∆∆Ct^ method was used to analyze the gene expression in the tissues [42].

## 3. Results

### 3.1. Growth and Survival

**Figure 2:**
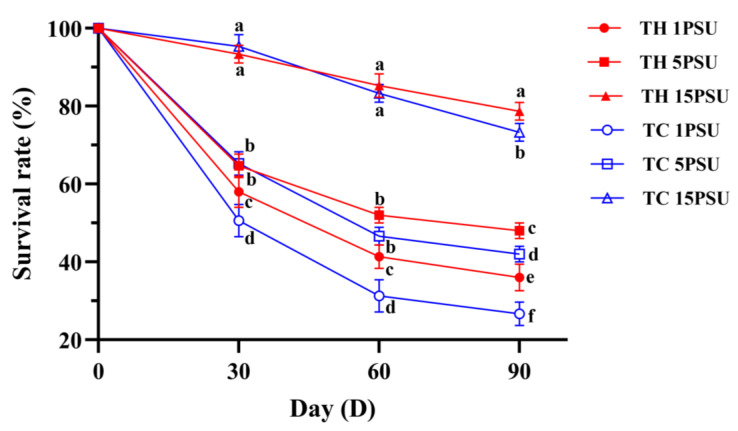
Survival rate of each group at different salinities at 30, 60, and 90 days. Different letters above the bars of the same series indicate significant differences (*p* < 0.05) among the different populations (mean ± SD, *n* = 3). TH, the low-salt breeding group; TC, the normal variety shrimp group.

**Figure 3:**
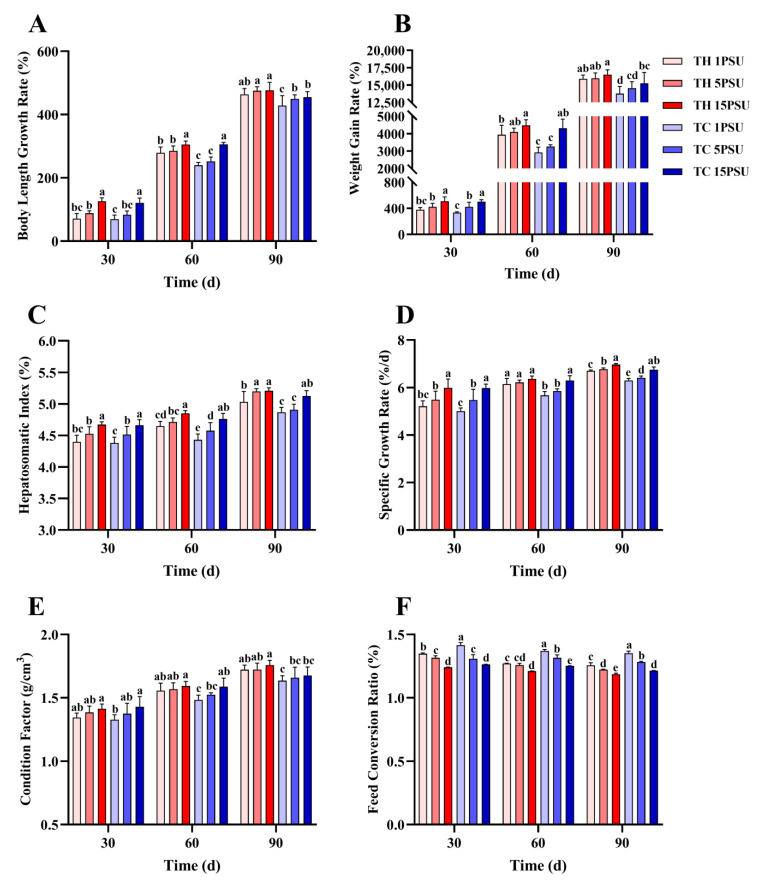
Growth parameters of each group at different salinities at 30, 60, and 90 days. (**A**) Body-length growth rate; (**B**) weight gain rate; (**C**) hepatosomatic index; (**D**) special growth rate; (**E**) condition factor; and (**F**) feed conversion rate. Different letters above the bars of the same series indicate significant differences (*p* < 0.05) among the different populations (mean ± SD, *n* = 9). TH, the low-salt breeding group; TC, the normal variety shrimp group.

Survival rates declined throughout the 90-day breeding period (Figure 2). However, at 90 days, the survival rate of the low-salt breeding group (TH group) was significantly higher than that of the normal variety shrimp group (TC group) at all salinities (*p* < 0.05). The growth performance showed that there was no significant difference (*p* > 0.05) in BGR (Figure 3A), WG (Figure 3B), or CF (Figure 3E) between the TH and TC on day 30, but at low salinities of 1 PSU and 5 PSU on day 60 and at all salinities on day 90, the scores of the TH were significantly higher (*p* < 0.05) than those of the TC. The HSI (Figure 3C) and SGR (Figure 3D) of TH were significantly higher (*p* < 0.05) than those of TC at low salinities of 1 PSU and 5 PSU on day 60 and day 90. The FCR (Figure 3F) of the TC was significantly higher (*p* < 0.05) than that of the TH at all salinities on days 60 and 90.

### 3.2. Biochemical Parameters of the Hepatopancreas and Muscle

**Figure 4:**
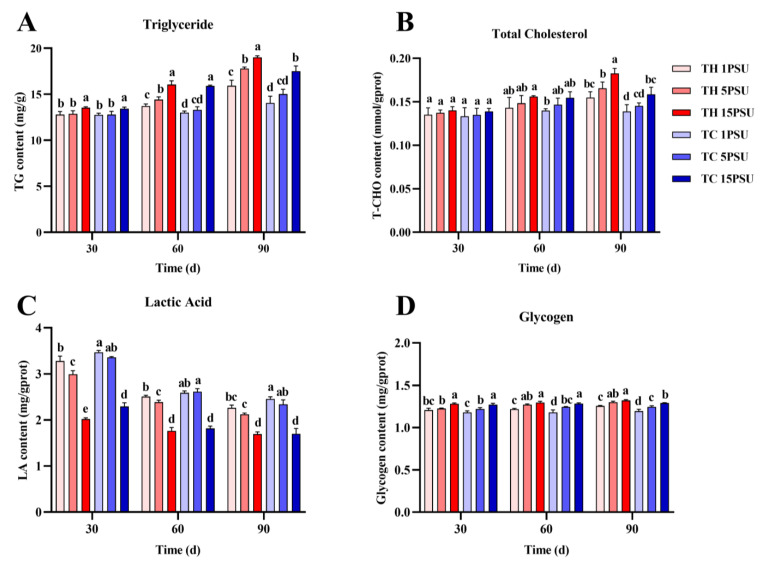
The levels of biochemical components in the hepatopancreas from each group at 30, 60, and 90 days. (**A**) Triglyceride; (**B**) total cholesterol; (**C**) lactic acid; and (**D**) glycogen. Different letters above the bars of the same series indicate significant differences (*p* < 0.05) among the different populations (mean ± SD, *n* = 3). TH, the low-salt breeding group; TC, the normal variety shrimp group.

**Figure 5:**
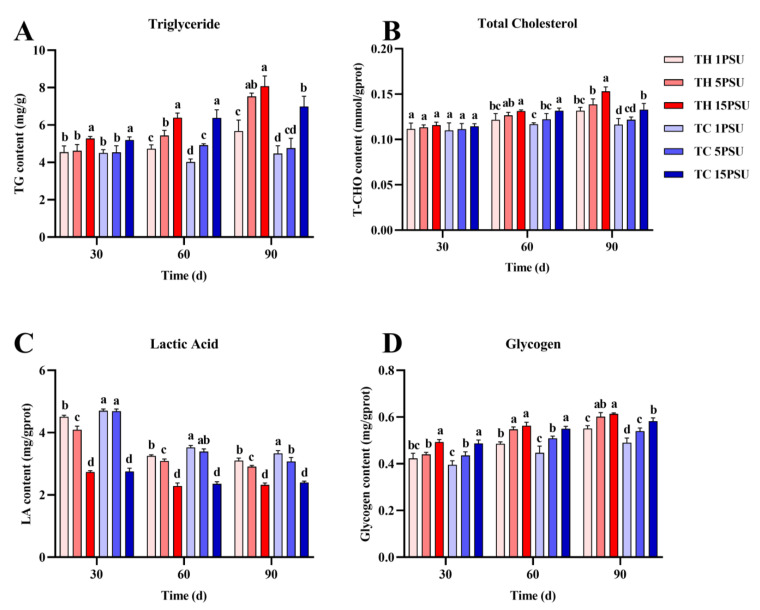
The levels of biochemical components in the muscles of each group at 30, 60, and 90 days. (**A**) Triglyceride; (**B**) total cholesterol; (**C**) lactic acid; and (**D**) glycogen. Different letters above the bars of the same series indicate significant differences (*p* < 0.05) among the different populations (mean ± SD, *n* = 3). TH, the low-salt breeding group; TC, the normal variety shrimp group.

In the hepatopancreas (Figure 4), levels of triglycerides, total cholesterol, and glycogen were significantly higher in the TH than in the TC at 90 days at all levels of salinity (*p* < 0.05). On the contrary, lactic acid content was the opposite. During the whole culture process, the lactic acid content in TH was significantly lower than TC when the salinity was 1 PSU and 5 PSU (*p* < 0.05). In muscle (Figure 5), the changes and outcomes in levels of triglycerides, total cholesterol, lactic acid, and glycogen were similar to those in the hepatopancreas.

### 3.3. Digestive Enzymes in the Hepatopancreas and Muscle

**Figure 6:**
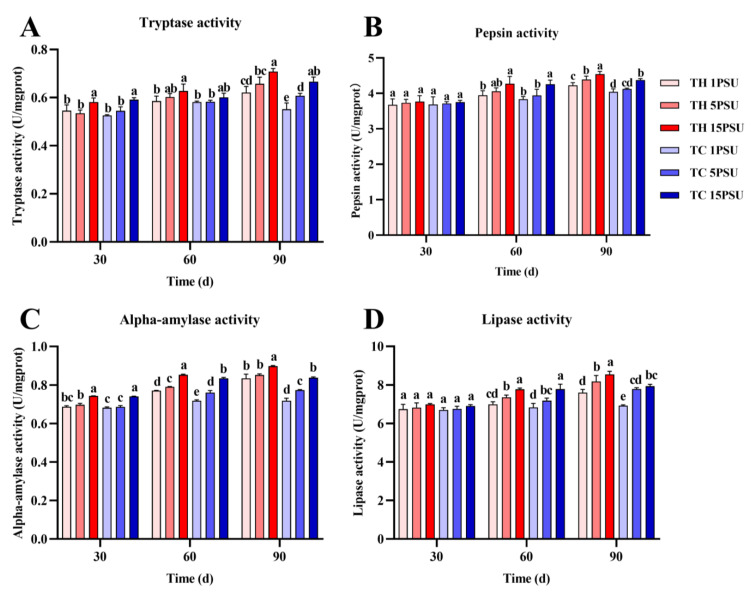
The activities of four digestive enzymes in the hepatopancreas of each group at 30, 60, and 90 days. (**A**) Tryptase activity; (**B**) pepsin activity; (**C**) alpha-amylase activity; and (**D**) lipase activity. Different letters above the bars of the same series indicate significant differences (*p* < 0.05) among the different populations (mean ± SD, *n* = 3). TH, the low-salt breeding group; TC, the normal variety shrimp group.

**Figure 7:**
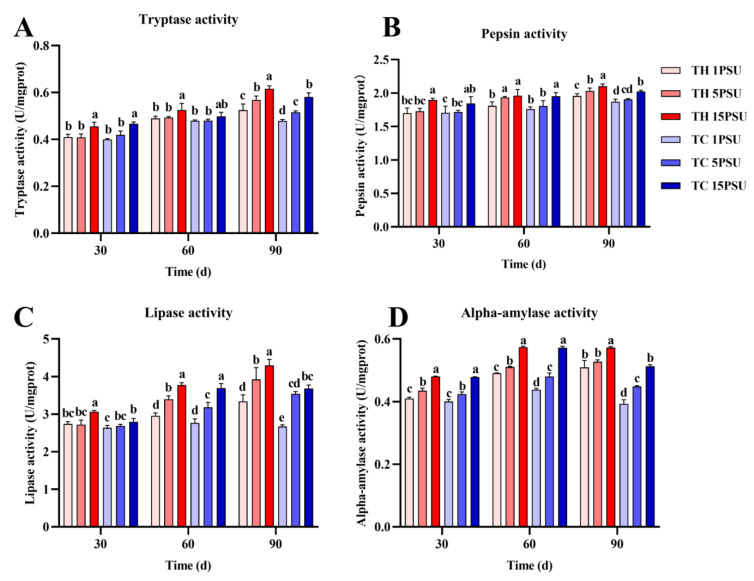
The activities of four digestive enzymes in the muscles of each group at 30, 60, and 90 days. (**A**) Tryptase activity; (**B**) pepsin activity; (**C**) alpha-amylase activity; and (**D**) lipase activity. Different letters above the bars of the same series indicate significant differences (*p* < 0.05) among the different populations (mean ± SD, *n* = 3). TH, the low-salt breeding group; TC, the normal variety shrimp group.

In the hepatopancreas (Figure 6), there was no significant difference in tryptase, pepsin, and lipase activity between the two groups of shrimps at 30 and 60 days. At 90 days, the activities of these three enzymes in TH were significantly higher than TC at 1 PSU and 5 PSU salinities (*p* < 0.05). As for the activity of alpha-amylase, TH was significantly higher than TC at 30 days in low salinities, and TH and TC were significantly different at 60 and 90 days in all salinities (*p* < 0.05). In muscle tissue (Figure 7), there was no significant difference in tryptase, pepsin, alpha-amylase, and lipase activities between the two groups of shrimp at 30 and 60 days. At 90 days, the activities of these enzymes in TH were significantly higher than TC in all salinities (*p* < 0.05).

### 3.4. Amino Acids and Fatty Acids Composition

After 90 days of culturing, we measured the amino acid content in muscle tissue of the TH and the TC at different salinities (Table 2). In general, glutamic (Glu) had the highest amino acid content, followed by aspartic (Asp). At low salinities of 1 PSU and 5 PSU, there were significant differences in total amino acid content and total essential amino acid content between TH and TC (*p* < 0.05), but there was no significant difference between the two groups at salinities of 15 PSU. At salinities of 1 PSU and 5 PSU, levels of the essential amino acids lysine (Lys) and isoleucine (Ile) in TH were higher than in TC. At a salinity of 1 PSU, the leucine (Leu) content in the TH was significantly higher than that in the TC. Among all treatment groups, TH at a salinity of 15 PSU had the highest glycine (Gly) content and the lowest proline (Pro) content.

Fatty acid composition (percentage of total fatty acids) was determined in hepatopancreas of different treatment groups after 90 days of culture (Table 3). In the present study, the highest proportion of SFA was palmitic acid (PA, C16:0), followed by stearic acid (SA, C18:0), which is consistent with previous reports [43]. Some SFAs, such as C15:0, C17:0, and C22:0, were significantly lower in the TH than in the TC (*p* < 0.05). Most MUFAs and PUFAs were not significantly different between either group. However, the HUFAs content of the hybrid population was significantly higher than that of the control population (*p* < 0.05), especially for docosahexaenoic acid (DHA, C22:6n-3). Overall, there was no particularly large difference in SFAs, MUFAs, and PUFAs (*p* > 0.05), whereas HUFA was shown to be significantly higher in the hybrid population than in the control population (*p* < 0.05).

### 3.5. Growth-Related Gene Expression

**Figure 8:**
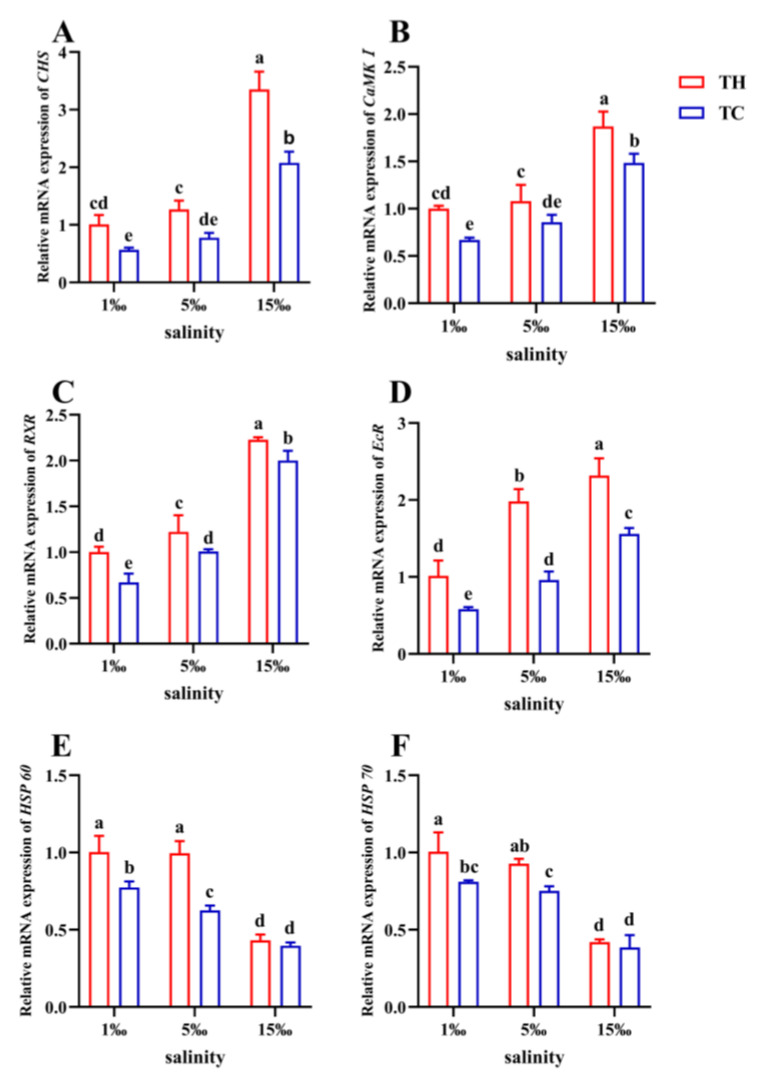
Molting-related genes expression of each group. (**A**) *CHS*, chitinase; (**B**) *CaMKI,* calmodulin-dependent protein kinase I; (**C**) *RXR,* retinoid X receptor; (**D**) *EcR*, ecdysone receptor; (**E**) *HSP60,* heat-shock protein 60; (**F**) *HSP70*, heat-shock protein 70. Different letters above the bars of the same series indicate significant differences (*p* < 0.05) among the different populations (mean ± SD, *n* = 3). TH, the low-salt breeding group; TC, the normal variety shrimp group.

The gene expression levels of *CHS*, *CaMKI*, *RXR*, and *EcR* in TH were significantly higher than those in TC under the same salinity (Figure 8, *p* < 0.05). At a low salinity of 1 PSU and 5 PSU, the expression of *HSP60* and *HSP70* in TH was significantly higher (*p* < 0.05) than in TC. Although at 15 PSU salinity, the expression of *HSP60* and *HSP70* was different, it was not significant (*p* > 0.05).

## 4. Discussion

Inbreeding affects the reproductive characteristics of shrimp, including *P. vannamei* [14]. Genetic improvement through hybridization can solve the problems of germplasm degradation caused by inbreeding, intensive farming, and other reasons. The combination of desirable traits from both parents usually produces offspring with heterosis, such as faster survival, growth performance, reproductive ability, and disease resistance [1]. The genetic heterozygosity of wild populations has decreased under intensive fishing, and hybridization and purification breeding could give hybrids a stable and dominant profile [44]. By comparing a shrimp bred in low salinity with normal variety shrimp after three months of culture, it was found that the growth performance (SR, WG, BGR, HSI, CF, FCR) of the TH was better than that of the TC at low salinity of 1 PSU and 5 PSU (Figure 2 and Figure 3). The TH bred by hybridization and low-salt selection may inherit genetic advantages from the parent and, therefore, have a stronger growth performance [44].

The hepatopancreas are the metabolic organs of crustaceans and are involved in digestion, the absorption of nutrients, and the storage of energy [23]. Digestive enzymes are mainly secreted by the hepatopancreas, and the increase of digestive enzyme activity is conducive to the absorption and digestion of nutrients, thus promoting growth [45]. Pepsin and trypsin are important hydrolytic and digestive enzymes in shrimp [46]. Pepsin plays an important role in nutrient metabolism [47]. Trypsin is a serine protease that hydrolyzes peptide bonds on the carboxyl groups of lysine, arginine, and ornithine [48]. Pancreatic alpha-amylase is a key enzyme in the digestive process that promotes the breakdown of starch [49]. Lipases have a potential role in hydrolyzing triacylglycerides stored as fat bodies [50]. The digestive enzyme activity of the TH was significantly higher than that of the TC, which was consistent with the better growth performance of the TH at low salinity (Figure 6 and Figure 7). The improvement of digestive enzyme activity promoted the digestion and metabolism of the shrimp and was conducive to the accumulation of nutrients [51].

Triglycerides and total cholesterol are common lipids found in marine organisms that provide energy to meet metabolic needs when food is scarce [52]. The activity of lipase was higher in the TH (Figure 6D and Figure 7D), which was conducive to the digestion and metabolism of triglyceride and total cholesterol. In addition to lipids that provide energy, glycogen in muscles and hepatopancreas can also provide energy as an energy substrate under stress [53]. Under low salinity, the TH has a higher glycogen content (Figure 4D and Figure 5D), which allows it to tolerate the stress environment. Crustaceans tend to accumulate lactic acid in the hemolymph when they expend energy [54]. Gluconeogenesis takes place in hepatopancreatic islets with noncarbohydrate precursors such as lactic acid [55]. Under the effect of salinity and other stresses, lactic acid in prawns would be increased to obtain more energy through anaerobic metabolism [26]. In all salinities, the lactic acid content of the TH was lower than that of the TC (Figure 4C and Figure 5C), indicating that low-salt-breeding hybrids may promote better adaptability to the environment and not produce an excessive stress response and anaerobic metabolism. The improvement of lipase, protease, and other digestive enzymes can promote the digestibility of lipids, protein, and other nutrients in the body, which is conducive to the accumulation of nutrients in the shrimp body [56]. The results of digestive enzyme activity and biochemical composition analysis showed that the TH had higher digestive enzyme activity and more nutrient accumulation than the TC in the low-salt environment (Figure 4, Figure 5, Figure 6 and Figure 7). It was proved that hybridization could effectively improve the growth performance of *P. vannamei* under low salt and stimulate heterosis, which was similar to previous studies [22].

Digestion and utilization of amino acids are crucial in shrimp culture [28]. Appropriate lysine can reduce the oxidation of other amino acids by increasing the utilization rate of other EAA, thus improving the growth rate of Penaeus shrimp [57]. Leucine can improve the growth performance and muscle crude protein content of *P. vannamei* [30]. At salinity conditions of 1 PSU and 5 PSU, higher lysine in the TH may promote the absorption and utilization of essential amino acids, and leucine may promote muscle growth and fiber synthesis to increase muscle crude protein content (Table 2). Besides, compared to the other treatment groups, we found that the TH group cultured at 1 PSU had the highest total free amino acid content (Table 2). As an organic osmotic regulator, free amino acids accumulate in high concentrations without disturbing cellular structure and metabolic activities [58]. Therefore, we speculate that hybridization improves the osmotic regulation ability of *P. vannamei* under low salt, so that it can grow better in a low-salt environment.

The fatty acid composition of shrimp is very important for individual growth and development and as an exogenous fatty acid intake for humans. DHA and EPA requirements are well established for some marine organisms [59,60]. As a euryhaline species, the same is true of *P. vannamei* [61]. DHA is closely related to shrimp growth. Previous studies showed that *P. vannamei* larvae with excellent growth performance had high DHA content [62], which was similar to our results. The DHA content of the TH was significantly higher than that of the TC and was positively correlated with the results of previous studies on growth-related indicators (Table 3). Combined with the results of growth indicators, we speculated that HUFA content could promote the growth of *P. vannamei*, but the specific mechanism still needs to be further explored. In terms of amino acid and fatty acid content, the TH contained high levels of key growth substances, which may be closely related to its superior growth performance.

The molting process of shrimp is closely related to growth and reproduction [63]. The process is related to the ecdysteroid receptor (EcR), retinoid X receptor (RXR), and chitin synthase (CHS) of the shrimp [39]. In addition, calmodulin-dependent protein kinase I (CaMKI) has been shown to have a molt-specific function in crustaceans [64]. The interactions of HSP60 play a role in immunity and stress [65]. Heat-shock protein 70 (HSP70) is concerned with the antioxidant defense system of *P. vannamei* [26]. Comparing the expression of genes associated with molting and stress, the TH was higher than that of the TC at low salinities of 1 PSU and 5 PSU (Figure 8). This indicated that the increase of stress-related genes in the TH was conducive to shrimp molting, causing them to express more molting-related genes and show better growth performance than the TC in a low-salt environment. The gene expressions of hormone receptors, hair-removal-related enzymes, and regulatory elements were highly expressed in the hybrids, indicating that the growth advantage and growth performance of TH were consistent at the molecular level. The molting of crustaceans is conducive to muscle growth and WG. Although the expression of growth-related genes in muscle was not analyzed in this study, Li, Jiang, Chen, Liu, Huang, Tian, Huang, and Zhao [22] showed that growth-related genes were highly expressed in muscle of hybrid populations. Therefore, we hypothesized that the expression of growth-related genes in the muscle of the hybrid population was similar to that of hepatopancreas in this experiment.

## 5. Conclusions

In conclusion, this study showed that the survival rate, growth performance, and nutritional quality of a low-salinity-tolerant hybrid in a low-salt culture were better than that of normal variety shrimp. The higher activity of digestive enzymes in the TH was consistent with its better growth performance and was also associated with higher triglyceride, total cholesterol, and glycogen content. The low level of lactic acid indicated that anaerobic metabolism occurred less in the body and was better adapted to the environment. The high survival rate and excellent growth performance of the TH in a low-salt environment may be related to the higher expression of genes related to molting and stress, the higher amount of the essential amino acids Lys and Leu, and the high HUFA content. The higher essential amino acid content in the TH indicated that it had a higher nutritional value. This research for subsequent genetic breeding and shrimp culture provided a valuable reference.

## Figures and Tables

**Table 1:** Real-time PCR primer sequences used in this study.

Primer	Sequences (5′−3′)	GenBank No.
CaMKI-F	CATCATAGAATGGAGGGTA	KU601407.1
CaMKI-R	AGAAGTCTTGGCACAGAA	
EcR-F	TGTAATCTGGTCCTCCCT	KF234770.1
EcR-R	AATAACTGACGACGACTCTG	
CHS-F	CGCGACGAGTTACTTTAGCAGT	AF315689.1
CHS-R	CGGCGGTTACAACGAGAA	
RXR-F	CTGTTGGGTCTGAGTTGAG	KC347569.1
RXR-R	GGACAAAGGGAGATAAAGAA	
HSP60-F	ATAACTCCACGCCTGATC	FJ710169.2
HSP60-R	GCCAACAACACCAACGAA	
HSP70-F	ACTCAGCTCGAACTTACCC	AY645906.1
HSP70-R	ACCACCTACTCTGACAACCA	
β-actin-F	TCCATGCCCAGGAATGAG	AF300705.2
β-actin-R	GAGCAGGAGATGACCACCG	

**Table 2:** Composition of amino acids in muscle (dry weight).

Aminon Acid (g/kg)	TH 1 PSU	TH 5 PSU	TH 15 PSU	TC 1 PSU	TC 5 PSU	TC 15 PSU
Met ^1^	2.44 ± 0.02 ^a^	2.4 ± 0.15 ^ab^	2.14 ± 0.04 ^c^	2.36 ± 0.07 ^ab^	2.35 ± 0.07 ^ab^	2.23 ± 0.07 ^bc^
Lys ^1^	7.6 ± 0.14 ^a^	7.52 ± 0.5 ^ab^	6.49 ± 0.13 ^d^	7.17 ± 0.22 ^bc^	6.92 ± 0.18 ^cd^	6.73 ± 0.28 ^cd^
Val ^1^	3.65 ± 0.06 ^a^	3.63 ± 0.25 ^a^	3.1 ± 0.07 ^c^	3.51 ± 0.08 ^ab^	3.35 ± 0.09 ^bc^	3.27 ± 0.21 ^bc^
Ile ^1^	3.6 ± 0.04 ^a^	3.52 ± 0.2 ^a^	3.06 ± 0.06 ^c^	3.33 ± 0.11 ^bc^	3.23 ± 0.07 ^bc^	3.12 ± 0.17 ^c^
Phe ^1^	3.67 ± 0.04 ^a^	3.6 ± 0.26 ^a^	3.16 ± 0.05 ^b^	3.49 ± 0.07 ^ab^	3.48 ± 0.09 ^ab^	3.38 ± 0.19 ^ab^
Leu ^1^	6.79 ± 0.08 ^a^	6.58 ± 0.38 ^ab^	5.84 ± 0.1 ^c^	6.34 ± 0.25 ^bc^	6.05 ± 0.14 ^bc^	5.94 ± 0.3 ^c^
Thr ^1^	3.36 ± 0.04 ^a^	3.3 ± 0.22 ^a^	2.91 ± 0.02 ^c^	3.23 ± 0.07 ^ab^	3.04 ± 0.08 ^bc^	2.94 ± 0.12 ^c^
His	1.74 ± 0.08 ^a^	1.78 ± 0.18 ^a^	1.46 ± 0.04 ^b^	1.72 ± 0.05 ^ab^	1.66 ± 0.07 ^ab^	1.63 ± 0.14 ^ab^
Arg	8.34 ± 0.34 ^a^	7.8 ± 0.54 ^ab^	7.36 ± 0.2 ^b^	8.28 ± 0.36 ^a^	7.85 ± 0.37 ^ab^	7.14 ± 0.51 ^ab^
Asp ^2^	9.4 ± 0.13 ^a^	9.28 ± 0.43 ^a^	8.09 ± 0.11 ^c^	8.81 ± 0.28 ^bc^	8.53 ± 0.22 ^bc^	8.3 ± 0.36 ^bc^
Ser ^2^	3.22 ± 0.04 ^a^	3.1 ± 0.27 ^ab^	2.84 ± 0.04 ^c^	3.17 ± 0.07 ^ab^	2.96 ± 0.08 ^bc^	2.87 ± 0.09 ^bc^
Glu ^2^	14.96 ± 0.37 ^a^	14.46 ± 1.17 ^a^	12.68 ± 0.09 ^c^	14.47 ± 0.42 ^a^	13.69 ± 0.26 ^bc^	13.41 ± 1.03 ^bc^
Gly ^2^	7.83 ± 0.55 ^b^	7.33 ± 0.2 ^bc^	9.72 ± 0.72 ^a^	7.16 ± 0.71 ^c^	8.02 ± 0.9 ^b^	7.31 ± 0.09 ^bc^
Ala ^2^	5.81 ± 0.25 ^a^	5.68 ± 0.29 ^ab^	5.11 ± 0.12 ^c^	5.31 ± 0.08 ^bc^	5.14 ± 0.09 ^c^	5.05 ± 0.18 ^c^
Tyr ^2^	3.5 ± 0.04 ^a^	3.38 ± 0.22 ^a^	3.03 ± 0.05 ^b^	3.29 ± 0.13 ^ab^	3.21 ± 0.02 ^ab^	3.25 ± 0.19 ^ab^
Pro ^2^	6.48 ± 0.82 ^a^	6.53 ± 1.46 ^a^	3.38 ± 0.3 ^b^	6.18 ± 0.8 ^a^	5.57 ± 0.21 ^a^	6.3 ± 0.33 ^a^
W_TAA_	92.3 ± 1.37 ^a^	89.88 ± 5.75 ^ab^	80.33 ± 0.94 ^c^	87.85 ± 2.43 ^bc^	85.02 ± 2.51 ^bc^	82.75 ± 3.84 ^bc^
W_EAA_	31.11 ± 0.36 ^a^	30.54 ± 1.92 ^ab^	26.69 ± 0.44 ^d^	29.42 ± 0.84 ^bc^	28.42 ± 0.71 ^cd^	27.61 ± 1.33 ^cd^
W_SEAA_	10.08 ± 0.42 ^a^	9.58 ± 0.72 ^ab^	8.82 ± 0.22 ^b^	9.99 ± 0.34 ^ab^	9.51 ± 0.43 ^ab^	8.77 ± 0.65 ^ab^
W_NEAA_	51.2 ± 0.72 ^a^	49.77 ± 3.06 ^ab^	44.84 ± 0.47 ^c^	48.39 ± 1.27 ^bc^	47.12 ± 1.36 ^bc^	46.49 ± 1.95 ^bc^
W_EAA_/W_TAA_	0.34	0.34	0.33	0.33	0.33	0.33
W_EAA_/W_NEAA_	0.61	0.61	0.60	0.61	0.60	0.59

Note: W_SEAA_, whole semi-essential amino acids; W_EAA_, whole essential amino acid; W_TAA_, whole total amino acids; W_NEAA_, whole nonessential amino acids. In the same line, values without or with the same letter superscripts indicate no significant difference (*p* > 0.05), whereas different letter superscripts represent significant differences (*p* < 0.05). TH, the low-salt breeding group; TC, the normal variety shrimp group. ^1^ Essential amino acids. ^2^ Nonessential amino acids.

**Table 3:** Fatty acid composition (% total fatty acids) in the hepatopancreas of each *P. vannamei* group.

	TH 1 PSU	TH 5 PSU	TH 15 PSU	TC 1 PSU	TC 5 PSU	TC 15 PSU
C14:0	0.83 ± 0.056 ^a^	0.83 ± 0.032 ^a^	0.82 ± 0.058 ^ab^	0.88 ± 0.094 ^a^	0.77 ± 0.063 ^ab^	0.67 ± 0.051 ^b^
C15:0	0.76 ± 0.042 ^b^	1.10 ± 0.105 ^a^	0.36 ± 0.013 ^c^	1.16 ± 0.151 ^a^	1.20 ± 0.111 ^a^	0.49 ± 0.010 ^c^
C16:0	19.26 ± 0.329 ^a^	19.01 ± 0.995 ^ab^	17.65 ± 0.318 ^bc^	20.04 ± 0.522 ^a^	17.79 ± 0.606 ^bc^	16.53 ± 0.301 ^c^
C17:0	0.77 ± 0.020 ^b^	0.74 ± 0.024 ^b^	0.50 ± 0.016 ^b^	1.58 ± 0.355 ^a^	1.42 ± 0.123 ^a^	0.71 ± 0.077 ^b^
C18:0	4.54 ± 0.077 ^d^	7.31 ± 0.355 ^a^	7.02 ± 0.514 ^ab^	5.26 ± 0.486 ^cd^	7.00 ± 0.44 ^ab^	6.01 ± 0.699 ^bc^
C22:0	0.18 ± 0.009 ^c^	0.21 ± 0.024 ^cd^	0.21 ± 0.015 ^cd^	0.26 ± 0.017 ^a^	0.26 ± 0.004 ^a^	0.23 ± 0.023 ^ab^
C16:1	2.50 ± 0.083 ^c^	2.24 ± 0.046 ^c^	1.79 ± 0.218 ^d^	3.25 ± 0.238 ^b^	3.72 ± 0.140 ^a^	2.29 ± 0.175 ^c^
C17:1	0.20 ± 0.012 ^a^	0.20 ± 0.026 ^a^	0.21 ± 0.011 ^a^	0.18 ± 0.012 ^a^	0.18 ± 0.019 ^a^	0.17 ± 0.017 ^a^
C18:1n-9	25.05 ± 0.329 ^ab^	23.83 ± 0.415 ^bc^	22.85 ± 0.898 ^c^	24.61 ± 1.617 ^abc^	24.63 ± 0.091 ^abc^	25.95 ± 0.506 ^a^
C20:1n-9	1.69 ± 0.044 ^abc^	1.53 ± 0.028 ^c^	1.63 ± 0.094 ^bc^	1.57 ± 0.101 ^bc^	1.73 ± 0.087 ^ab^	1.87 ± 0.112 ^a^
C22:1n-9	0.38 ± 0.04 ^d^	0.49 ± 0.047 ^cd^	0.56 ± 0.07 ^abc^	0.51 ± 0.031 ^bcd^	0.68 ± 0.067 ^a^	0.67 ± 0.112 ^ab^
C18:2n-6	26.52 ± 0.328 ^a^	23.55 ± 0.811 ^c^	26.00 ± 0.923 ^ab^	25.61 ± 0.196 ^ab^	24.56 ± 0.222 ^bc^	25.62 ± 0.608 ^ab^
C18:3n-3	2.06 ± 0.111 ^ab^	1.14 ± 0.044 ^d^	1.82 ± 0.047 ^c^	1.94 ± 0.052 ^bc^	1.24 ± 0.138 ^d^	2.27 ± 0.090 ^a^
C20:2n-6	1.63 ± 0.127 ^b^	1.07 ± 0.139 ^e^	1.51 ± 0.091 ^bc^	1.34 ± 0.125 ^cd^	1.19 ± 0.048 ^de^	2.10 ± 0.111 ^a^
C20:3n-6	0.11 ± 0.007 ^b^	0.15 ± 0.004 ^a^	0.12 ± 0.005 ^b^	0.11 ± 0.008 ^b^	0.16 ± 0.005 ^a^	0.15 ± 0.012 ^a^
C20:4n-6	1.07 ± 0.104 ^d^	2.88 ± 0.037 ^a^	2.33 ± 0.268 ^b^	1.42 ± 0.049 ^d^	1.96 ± 0.109 ^bc^	1.89 ± 0.217 ^c^
C22:5n-3	6.27 ± 0.206 ^bc^	7.28 ± 0.223 ^a^	7.26 ± 0.318 ^a^	4.85 ± 0.057 ^d^	5.87 ± 0.390 ^c^	6.97 ± 0.503 ^ab^
C22:6n-3	6.2 ± 0.134 ^bc^	6.44 ± 0.183 ^b^	7.37 ± 0.399 ^a^	5.43 ± 0.278 ^d^	5.63 ± 0.152 ^cd^	5.42 ± 0.246 ^d^
SFAs	26.33 ± 0.282 ^bc^	29.21 ± 1.448 ^a^	26.56 ± 0.775 ^bc^	29.19 ± 1.459 ^a^	28.44 ± 0.664 ^ab^	24.64 ± 1.022 ^c^
MUFAs	29.81 ± 0.308 ^ab^	28.29 ± 0.381 ^bc^	27.03 ± 1.215 ^c^	30.12 ± 1.524 ^ab^	30.95 ± 0.289 ^a^	30.94 ± 0.605 ^a^
PUFAs	28.58 ± 0.428 ^a^	24.69 ± 0.836 ^b^	27.81 ± 0.935 ^a^	27.54 ± 0.179 ^a^	25.80 ± 0.318 ^b^	27.89 ± 0.544 ^a^
HUFAs	15.28 ± 0.281 ^c^	17.82 ± 0.234 ^a^	18.59 ± 0.518 ^a^	13.15 ± 0.364 ^d^	14.81 ± 0.398 ^c^	16.53 ± 0.654 ^b^

Note: SFAs: C14:0, C15:0, C16:0, C17:0, C18:0, C22:0; MUFAs: C16:1, C17:1, C18:1n-9, C20:1n-9, C22:1n-9; PUFAs: C18:2n-6, C18:3n-3; HUFAs: C20:2n-6, C20:3n-3, C20:4n-6, C22:5n-3, C22:6n-3. In the same line, values without or with the same letter superscripts indicate no significant difference (*p* > 0.05), whereas different letter superscripts represent significant differences (*p* < 0.05). TH, the low-salt breeding group; TC, the normal variety shrimp group.

## Data Availability

The authors declare that all data supporting the conclusions of this study are available within the article.
